# Fucanomics and Galactanomics: Marine Distribution, Medicinal Impact, Conceptions, and Challenges

**DOI:** 10.3390/md10040793

**Published:** 2012-03-29

**Authors:** Vitor H. Pomin

**Affiliations:** Program of Glycobiology, Institute of Medical Biochemistry, and University Hospital Clementino Fraga Filho, Federal University of Rio de Janeiro, Rio de Janeiro, RJ, 21941-590, Brazil; Email: vhpomin@gmail.com; Tel.: +55-21-2562-2939; Fax: +55-21-2562-2010

**Keywords:** fucanome, fucanomics, galactanome, galactanomics, glycome, glycomics, sulfated fucan, sulfated galactan

## Abstract

Glycomics turned out to be a very extensive project where its subdivision is consequently emerging. This is seen by the growing number of terminologies used to define subprojects concerning particular classes of bioactive carbohydrates. Sulfated fucans (SFs) and sulfated galactans (SGs) are relatively new classes of sulfated polysaccharides (SPs) that occur mostly in marine organisms, and exhibit a broad range of medicinal effects. Their structures are taxonomically dependent, and their therapeutic actions include benefits in inflammation, coagulation, thrombosis, angiogenesis, cancer, oxidation, and infections. Some red algae, marine angiosperm and invertebrates express SPs of unique structures composed of regular repeating oligomeric units of well-defined sulfation patterns. This fine pattern of structural regularity is quite rare among any naturally occurring long SPs, and enables accurate structure-biofunction correlations. Seeing that, fucanomics and galactanomics may comprise distinguished glycomics subprojects. We hereby discuss the relevance that justifies the international recognition of these subprojects in the current glycomics age associated with the beneficial outcomes that these glycans may offer in drug development.

## 1. Fucanome and Galactanome: Getting into Glycomics

After many “ome” projects such as genome, metagenome, transcriptome, proteome, lipidome and metabolome, glycome has ultimately launched itself into the world of biology. Glycome is the specific project that deals with carbohydrates or glycosylated molecules [1]. It is generally defined as a set of glycans or glycoconjugates expressed by a living form (cell, tissue, organ, or organism) under certain circumstances [1]. Likewise, glycomics is defined as the comprehensive structural and functional studies of glycome(s) of individual or group of organisms [1,2]. Based on clear evidences, glycomics are so far bringing more challenges than the first “omics” [3]. This is particularly due to: (i) higher structural complexity and the flexibility of glycans, which consequently make data interpretation more difficult [3,4]; (ii) lack of a clear automatic sequencing method for complex carbohydrates as opposed to those routinely employed for nucleic acids (in genomics) and proteins (in proteomics) [3]; (iii) existence of innumerable, possible determinants in the biosynthesis of glycans which usually allows multiple questionings [1,2,3]; and (iv) existence of many sub-classes of glycans or glycoconjugates [3,5].

Because of these generally overwhelming challenges, a tendency to undertake or provide sectorized emphasis into sub-classes of glycans has consequently and naturally emerged in glycomics [3,5,6,7,8,9,10,11,12,13]. Empirical subprojects defined by novel terminologies are spreading across the community, and they aim to deal essentially with particular sub-classes of bioactive carbohydrates. Recent nomenclatures that are coming into common use are Proteoglycanome [6], Glycosaminoglycanome [7], Heparanome [8,9,10], Glycolipidome [13], Glycoproteome [12], and Sialome [11]. Glycomics seems perhaps to be ultimately evolving more as a conjunction of isolated or individualized works [3,5,11,14] rather than just a single compact ongoing project. The propensity to adopt such subdivisions and terminologies seems to be less evident than in the other “omics”.

Sulfated fucans (SFs) and sulfated galactans (SGs) are classes of sulfated polysaccharides (SPs) composed essentially of α-L-fucopyranosyl (Fuc*p*), or α-L-, α-D-, β-D-galactopyranosyl (Gal*p*) units, respectively. SFs and SGs are distinct from the majority of other glycans in the following aspects:

(i) relatively young and short literature timeline;
(ii) basically found in marine organisms in which the structures are taxonomically related;
(iii) fine and very rare patterns of structural regularity in certain cases;
(iv) broad range of therapeutic actions with high levels of effectiveness in certain assays;
(v) well-defined SFs and SGs achieve advanced structure-biofunction correlations more easily.

In accordance with the actual tendency for assuming subprojects in the current glycomics age, and due to the five above-cited features, we herein propose the dissemination of the terms Fucanome and Galactanome, and of the respective projects Fucanomics and Galactanomics, each concerning SFs and SGs, respectively. These SPs have gained some international recognition and are now under investigation in laboratories of many countries. From now on, we hereby discuss in detail the main characteristics of SFs and SGs that justify the investments into their research in the current glycomics age. We also give appropriate arguments for international assimilation of such projects into a closer collaborative network, and suggested topics for discussion in future glycoscientific events.

### 1.1. SFs and SGs: Young Molecules of Glycobiology

The bulk interest in structural and biological studies of SFs and SGs effectively began in the early 90’s [15]. This particularly results from the worldwide advancement of glycobiology, particularly due to the dissemination and progress of powerful analytical tools such nuclear magnetic resonance (NMR) spectroscopy and mass spectrometry (MS), associated with the promising medicinal properties of SFs and SGs [15,16,17,18,19,20,21]. Research from some two decades has not been enough to make these SPs as internationally prominent as other bioactive carbohydrate classes, such as sialylated glycans or glycosaminoglycans (GAGs). Even though the SFs and SGs may show distinguished structural and biomedical properties, as discussed here, the latter classes are more widely recognized because of their natural physiological roles in humans. Specific terms therefore arose to define particular subprojects of these latter glycans: Sialomics [11], and Glycosaminoglycanomics [7], respectively. An even hierarchically lower subproject of Glycosaminoglycanomics, and in turn of Proteoglycanomics, have appeared: Heparanomics, which aims to deal with the bioactive domain of heparan sulfates [3,6,8,9,10]. The similar use of Fucanomics and Galactanomics still remain, but shall be disseminated across the international glyco-society, because of the uniqueness in both structures and functions of SFs and SGs.

### 1.2. Marine Taxonomic Distribution of SF and SG Structures

The richest sources of SFs and SGs are marine organisms, and their lack of occurrence in terrestrial mammals, especially humans, is two of the reasons for the narrow international fame of these glycans. Considering only the marine environment, SFs and SGs are expressed by macroalgae, marine angiosperms and invertebrates [16]. However, few outside works have reported the occurrence of SGs in bacteria and fungi as well [22]. Virtually ubiquitous in cell walls of all macroalgae, SFs have been synthesized in brown seaweeds (Phaeophyceae), in which they are also commonly known as fucoidans [16,18,19,20]. Whereas in green (Chlorophyceae) and red seaweeds (Rhodophyceae) only SGs have been found so far [16,19,21]. However, SFs and SGs can both be found in invertebrate animals, such as ascidians (also known as sea squirts or tunicates) (Urochordata, Ascidiaceae), sea cucumber (Echinodermata, Holothuroidea) and sea urchins (Echinodermata, Echinoidea) [16,20,21]. In ascidians and sea cucumbers, these SPs are known to participate in the structural assemblies of their body walls [15], most probably assembling the cell walls in macroalgae. In sea urchins, these SPs have been found building the jelly coat that surrounds the female gametes, and are known to participate in species-recognition during the initial steps of the fertilization process of these animals [15]. Marine angiosperms (Angiospermae, Spermatophyta) are another potential source for SGs, as evidenced by the work of Aquino *et al.* [23]. However, the lack of additional reports compromises the definitive assertion of marine superior plants as a confident source for this material. Again, as in algae, the angiosperm SGs contribute to build-up of cell walls in different tissues [24]. With no exceptions so far, SFs and SGs have unequivocally been shown to exist as essential components of the extracellular matrices in these marine organisms [16].

The structures of SFs and SGs are directly dependent on the species in which they occur, but some general aspects are still restricted to the phyla [15,16]. SFs from brown algae are usually the most complex molecules, even though mostly consisting of L-Fuc*p* units. The presence of other monosaccharide types associated with occasional sparse branches enhances structural complexity. The occurrence of repetitive units in brown algal SFs is somewhat still uncertain, but evidence supporting such a concept has appeared along the past few years, at least in certain species [16]. This is most likely a consequence of the advances in instrumentation and methods capable for the structural analysis of complex carbohydrates. Nonetheless, the currently proposed oligomeric repeating motifs of certain fucoidans still show high degrees of heterogeneity (Table 1). Regardless of structural patterns, brown algal SFs are the most abundant SPs in the sea, and perhaps across the entire globe, since brown seaweeds by far dominate the sea environment in both number of species (1.5 to 2 thousand) and biomass [16], as the sea environment totals more than two-thirds of the planet.

**Table 1:** Illustrative examples of repetitive units currently assumed for famous phaeophyceae species.

Brown Seaweed Species	Proposed Repetitive Structure	Ref.
*Ascophylum nodosum*	[→4-α-L-Fuc*p* -2,3di(SO_3_^−^)-1→3-α-L-Fuc*p*-2(SO_3_^−^)-1→]_n_ + branches of non-sulfated α-L-Fuc*p*	[24]
*Fucus evanescences*		[25]
*Fucus vesiculosus*		[24]
*Ecklomia kurome*	[→3-α-L-Fuc*p*-2R,4(SO_3_^−^)-1→]_n_, where R = H or SO_3_^−^	[26]
*Chorda filum*	[→3-α-L-Fuc*p*-2R^1^,4R^2^-1→3-α-L-Fuc*p*-2R^1^,4R^2^-1→3-α-L-Fuc*p*-2R^1^,4R^2^-1→3-α-L-Fuc*p*-2(→1-α-L-Fuc*p*-2,4diR^2^), 4R^2^-1→3-α-L-Fuc*p*-2R^1^,4R^2^-1→]_n_, where R^1 ^= H or SO_3_^−^ or COCH_3_, and R^2^ = H or SO_3_^−^	[27]

The discovery of SGs in green algae is quite recent. *Codium *has been the genus most studied so far [15,28,29,30]. Green algal SGs usually show structures that are much less complex than those of brown algal SFs, but still more complex than those of red algal SGs, as discussed below [16,21]. Although no clear evidence that regular sequences really exist in green algal SG backbones, some clues have favored the concept of chains predominantly composed of 4-sulfated 3-linked β-D-Gal*p *units [15,29,30]. However, these chains may still bear high degrees of heterogeneity, like pyruvylated non-reducing terminal residues, and occasional branches. Sulfation positions other than those at C4 may also occur and increase complexity as well [29,30].

Among the three macroalgal classes, red seaweeds are most likely the only class able to really express SPs in regular backbones [21]. Like GAGs, red algal SG backbones are normally composed in disaccharide repeating units, but of alternating 3-linked β-D-Gal*p* and 4-linked α-D- or α-L-Gal*p* units [16,21]. The possible presence of an extra-bond between C3 and C6 of the same ring leads to the 3,6-anhydro-Gal*p* (3,6-AnGal*p*) unit that can occur only at the 4-linked Gal*p* unit. The enantiomeric variation, D- or L-, in this 4-linked unit respectively results in the nomenclature “carrageenan” or “agaran”. The names carrageenose or agarose are respectively related to these molecules when 3,6-AnGal*p* units occur along them [21]. Sulfated esters and/or occasionally methyl esters may occur at the 2- and/or 4-position(s) of the 3-linked Gal*p* units. These same substituents may be placed at 2-, 3-, and/or 6-position(s) of the 4-linked Gal*p* units as well. All these structural variations comprise the main heterogeneities in red seaweed SGs. But since the sugar chains of these polymers are regularly composed of repeating disaccharides, the difficulties in structural characterization are significantly diminished, compared to those from the other algal classes. In works concerning structural characterization of red algal SGs, these glycans have usually been extensively characterized, generally through a combination of NMR spectroscopy, particularly ^13^C-based spectra, with data analysis generated from chemical reactions [31,32,33].

### 1.3. Rare Structural Regularity among Polysaccharides of High-Molecular Weights

According to what has been stated before, the structural simplicity in macroalgal SPs rises in the following order: brown algal SFs, green algal SGs and red algal SGs. But even though red algae express SGs in disaccharide repeating units, there are still certain degrees of heterogeneity that impairs the arrival of a totally regular structural design. However, most likely through the steps of evolution, this complete structural pattern of regularity became noticeable in SPs from superior plants and from some marine invertebrates [21,23], probably due to a more organized or even simpler biosynthetic machinery in these latter organisms [15,16].

Table 2 depicts some illustrative examples of SF and SG of well-defined structures. It is clearly seen that the structures occur through a species-specific manner, varying in sulfation patterns (but always restricted to 3-*O*-, 2-*O*- and/or 4-*O*-positions), in glycosidic linkages [α(1→3), α(1→4), and β(1→3)], in repetitive oligomeric lengths (tetrasaccharides, trisaccharides, disaccharides, and monosaccharides), and sometimes in the presence of short branching segments such as the structure of *Styela plicata*, but still conserving the fine pattern of regularity. The molecular weight (MW) of these polymers, although quite polydisperse, are commonly very high, frequently ranging above 100 kDa. In polymers composed of a repetitive tetrameric unit as observed for echinoderms *Ludwigothuria grisea *and *Lytechinus variegatus *(Table 2), the chain extension of such glycans would range approximately over 100 tetrameric units. Certain red algae express SGs with quite regular structures, and thus must be gathered at the list of Table 2 as well. Overall, these fine patterns of regularity are very rare among naturally-occurring long polysaccharides.

**Table 2:** Oligomeric repetitive units of SFs and SGs from the echinoderms: sea urchins (Echinoidea), and sea cucumber (Holothuroidea); from red algae (Rhodophyta): marine superior plant (Angiospermae), and ascidians, also known as tunicates (Ascidiacea).

Species (Class)	Structure	Occurrence	Ref.
*Ludwigothuria grisea *(Holothurioidea)	[→3)-α-L-Fucp-2,4(OSO_3_^−^)-(1→3)-α- L-Fuc *p*-(1→3)-α- L-Fuc*p*-2(OSO_3_^−^)-(1→3)-α- L-Fuc*p-*2(OSO_3_^−^)-(1→]_n_	Brazil	[34]
*Strongylocentrotus purpuratus* II (Echinoidea)	[→3)-α-L-Fuc *p-*2,4di(OSO_3_^−^)-(1→3)-α- L-Fuc*p-*4(OSO_3_^−^)-(1→3)-α- L-Fuc*p-*4(OSO_3_^−^)-(1→]_n_	USA	[35]
*Strongylocentrotus purpuratus* I (Echinoidea)	80% [→3)-α-L-Fuc *p-*2,4di(OSO_3_^−^)-(1→]_n _and 20% [→3)-α-L-Fuc*p-*2(OSO_3_^−^)-(1→]_n_	USA	[35]
*Strongylocentrotus franciscanus *(Echinoidea)	[3)-α-L-Fucp-2(OSO3−)-(1→]n	USA	[36]
*Strongylocentrotus droebachiensis *(Echinoidea)	[→4)-α-L-Fuc *p-*2(OSO_3_^−^)-(1→]_n_	USA, Norway	[37]
*Strongylocentrotus pallidus *(Echinoidea)	[→3)-α-L-Fuc *p-*2(OSO_3_^−^)-(1→3)-α- L-Fuc*p-*2(OSO_3_^−^)-(1→3)-α- L-Fuc*p*-(1→3)-α- L-Fuc*p*-(1→]_n_	USA, Norway	[37]
*Lytechinus variegatus *(Echinoidea)	[→3)-α-L-Fuc *p-*2(OSO_3_^−^)-(1→3)-α- L-Fuc*p-*2(OSO_3_^−^)-(1→3)-α- L-Fuc*p-*4(OSO_3_^−^)-(1→3)-α- L-Fuc*p-*2,4di(OSO_3_^−^)-(1→]_n_	Brazil	[34]
*Arbacia lixula *(Echinoidea)	[→4)-α-L-Fuc *p-*2(OSO_3_^−^)-(1→4)-α- L-Fuc*p-*2(OSO_3_^−^)-(1→4)-α- L-Fuc*p*-(1→4)-α- L-Fuc*p*-(1→]_n_	Brazil	[38]
*Echinometra lucunter *(Echinoidea)	[→3)-α-L-Gal *p-*2(OSO_3_^−^)-(1→]_n_	Brazil	[38]
*Glyptosidaris crenularis *(Echinoidea)	[→3)-β-D-Gal *p-*2(OSO_3_^−^)-(1→3)-β-D-Gal*p*-(1→]_n_	Japan	[39]
*Botryocladia occidentalis* (Rodophyta)	[→3)-β-D-Gal *p-*2R_1_-3R_2_-(1→4)-α-L-Gal*p-*2R_3_-3R_4_-(1→]_n_, where R_1-4_ = OSO_3_^−^or OH, R_1_ and R_2_ = OSO_3_^−^ in ~66%, and ~33%, respectively.	Brazil	[40]
*Gelidium crinale *(Rodophyta)	[→3)-β-D-Gal *p-*2R_1_-4R_2_-(1→4)-α-L-Gal*p-*2R_3_-3R_4_-(1→]_n_, where R_1-4_ = OSO_3_^−^or OH, R_1_ and R_2_ = OSO_3_^−^ in ~60%, and ~15%, respectively.	Brazil	[41]
*Gigartina skottsbergii *and *G. chamissoi *(Rodophyta)	[→3)-β-D-Gal *p-*2R_1_-4R_2_-(1→4)-α-D-Gal*p-*2R_3_-6R_4_-(1→]_n_, where λ-carrageenan R_1_, R_3_ = OSO_3_^−^and R_2_, R_4_ = OH; µ-carrageenan R_1_, R_3_ = OH, and R_2_, R_4_ = SO_3_^−^; ν-carrageenan R_1 _= OH and R_2_, R_3_, R_4_ = SO_3_^−^; κ-carrageenan R_1_, R_3_ = OH and R_2_ = OSO_3_^−^; ι-carrageenan R_1_ = OH and R_2_, R_3_ = OSO_3_^−^. κ- and ι-carrageenans have 4-linked α-D-Gal*p* units cyclized as 3,6-anhydro: whereas µ- and ν- are just partially cyclized.	Argentina, others	[42,43]
*Rupia maritma *(Angiospermae)	[→3)-β-D-Gal *p*-2(OSO3^−^)-(1→4)-α-D-Gal*p*-(1→4)-α- D-Gal*p*-(1→3)-β-D-Gal*p*-4(OSO3−)-1→]_n_	Brazil	[23]
*Styela plicata *(Ascidiacea)	{→4)-α-L-Gal *p-*2[→1)-α-L-Gal*p*]-3(OSO_3_^−^)-(1→}_n_	Brazil	[44]
*Hedmania monus *(Ascidiacea)	[→4)-α-L-Gal *p-*3(OSO_3_^−^)-(1→]_n_	Brazil	[45]

### 1.4. The Impressively Wide Range of Medicinal Effects of SFs and SGs

Besides the natural actions of SFs and SGs in their own organisms of occurrence, these glycans have shown potential medicinal effects. In fact, they have been targets of research by many international groups, strictly due to their therapeutic promise. As the SFs and SGs consist of highly negatively-charged molecules, they have biophysical capacity to electrostatically interact with many health/disease-related basic proteins, or with positively-charged structural assemblies, such as the positive regions of virus particles. Although these electrostatic interactions depend essentially on a difference of net charge, such as sulfation density *vs.* basic amino acid content, it has been clearly proven in the past few years that the molecular interactions involving SFs and SGs are, in fact, mainly stereospecific (directly dependent on sulfation and glycosylation sites, anomericity, and monosaccharide-type composition), and not a mere consequence of the bulk charges related to sulfation degrees [15]. We briefly present below the general biochemical mechanisms of SFs and SGs in their main studied medical actions. It is noteworthy that the definitive mechanisms underlying the biomedical properties of these glycans are still under investigation.

#### 1.4.1. Inflammation

In anti-inflammatory actions, possible mechanisms have been postulated by which SFs and SGs may affect the leukocyte recruitment to sites of injury where inflammation normally evolves [46]. The ability of SFs and SGs to prevent selectin-mediated cell-cell interactions was successfully evidenced by some *in vitro* experiments using brown algal SFs [46]. These molecules have proved to bind directly to purified and membrane-exposed P- and L-selectins [46,47], but curiously not to E-selectins [48]. The interaction of SFs and SGs with specific selectins points toward the concept of a selective beneficial action of these glycans in inflammation, and consequently supports the advantages of selectivity in drug candidates. The binding of these glycans at selectins therefore impairs the consequential selectin-mediated cell migration of the activated leukocytes to go further to the specific sites of injury. There is also a current hypothesis that exogenous SPs, like marine SFs and SGs, might compete with GAG molecules from cell surface proteoglycans for cytokine/chemokine bonds. This consequently arrests the correct formation of cytokine/chemokine gradients necessary for leukocyte activation and migration.

#### 1.4.2. Hemostasis and Vascular Biology

Disorders related to the cardiovascular system and hematology, are the major cause of human death, accounting for around one-third of the total. Despite many downsides, GAG-type heparin is the most exploited biomolecule for treating these disorders. Heparin has a narrow therapeutic window and a highly variable dose-response relationship, necessitating frequent coagulation monitoring during its administration. The main side-effects of this drug are hemorrhage, thrombocytopenia and osteoporosis. Moreover, the heparin sources utilized for large-scale production are very limited. It is obtained from pig intestine or bovine lung, and contamination of samples with pathogens is a serious concern in the extraction procedure from these sources [49,50]. The situation was further complicated recently due to contamination of heparin preparations with over-sulfated chondroitin sulfate [51]. This contaminant is severe since it induces hypotension associated with kallikrein release when intravenously injected [52].

New alternatives for treatments of diseases related to the cardiovascular system are therefore urgently required, and, in addition, SFs and SGs also display efficient levels in anticoagulation [15,16,49,50]. In fact, it is these marine glycans’ biomedical effects as anticoagulants and antithrombotics that have been the most studied so far [15,16,46,49,50]. Their mechanisms of action most likely resemble the anticoagulant action of heparin, which potentiates the catalytic rates of natural blood inhibitors: the serpins antithrombin and heparin cofactor II [15,16,49,50,53,54]. However, in addition to this action, a serpin-independent anticoagulant mechanism was recently discovered for a red algal SG [55]. This novel mechanism is still unclear, but points toward a concept of the ability of this SG to blocking intermolecular complexes involving blood co-factors in initial steps of the coagulation cascade, such as the intrinsic pro-coagulant tenase and protrombinase complexes [55].

#### 1.4.3. Angiogenesis

SFs and SGs have shown the ability to inhibit the formation of neovascularization, mainly by interfering in the necessary binding of molecules responsible for angiogenesis, such as vascular endothelial growth factors (VEGFs) [46,56], and/or basic fibroblast growth factors (bFGFs) [46,57], to their respective receptors. These molecular interventions have proved of huge clinical interest, especially for inhibiting the consequential feeding of tumor-affected areas in cancer-committed patients. In the report of Cumashi and co-workers [46], through a systematic and comparative tubulogenic assay using human umbilical vein endothelial cell (HUVEC) in culture, incubated with fucoidans of different structures, it has been shown that the angiogenic effects of brown algal SFs are highly stereospecific. The antiangiogenic effects of a green algal SG (again genus *Codium*) have also been demonstrated [58].

#### 1.4.4. Tumor Progression and Spreading

The clearest mechanisms of SPs as potential candidates in anti-tumor therapies are most likely related to the inhibitory action of these glycans on tumor vascular networks, as evidenced by reduced rates in hemoglobin content in the affected areas using fucoidans [59]. Besides this neovascularization inhibiting function, fucoidans have proved to synergically reduce tumor spreading. This was observed through *in vitro* assays of cell-adhesion using highly metastatic breast cancer cell lines capable of binding normally onto human platelets fixed in a Matrigel. In some tests in which differential fractions of fucoidans were added, the binding property of the tumor cells can be abolished or reduced [59]. The ability of certain fucoidan-derivatives to interfere with breast cancer cell adhesion onto platelet-coated surfaces favors the concept that specific internal bioactive structural motifs of brown algal SFs really exist, and are responsible for combating metastasis [59]. This same experiment type was carried out using nine different fucoidans and heparin [46]. The authors observed that SFs from five specific brown algal species were able to reduce approximately 80% of the cell adhesion of tumor cells onto human platelet-coated plates [46]. The effectiveness reached by these fucoidans in tumor cell-adhesion was significant, and even higher than that achieved by highly sulfated glycans such as heparin, corroborating again the structural specificity in such clinical actions, rather than just a consequence of simple net charges.

#### 1.4.5. Antioxidation

Recent reports have pointed out the ability of algal SGs to reduce oxidation levels through *in vitro* assays that might resemble the mechanisms of oxidative stress in mammals [60,61,62,63,64]. These reports have shown that these marine SPs are capable of inhibiting hydroxyl and superoxide radical formation or to chelate oxidant iron ions such as Fe(II), thus impairing their prompt oxidative effects in metabolisms, and consequently preventing their damaging effects on health. The primary antioxidant action of these glycans seems to be notably dependent on their charge nature, and future work must be done to prove the possible structural or conformational behavior of these SPs in such activities. The description of antioxidative effects of these marine SPs heavily corroborates the concept of Fucanomics and Galactanomics as promising projects of glycomics in terms of their medicinal impact, but, above all, supports the consumption of marine algae as food supplements—mainly because of the beneficial nutraceutical components contained in these organisms, such as antioxidants.

#### 1.4.6. Infections

Infections and parasitic diseases are the second largest cause of death in humans, accounting for around one-fourth of the total. SFs and SGs have shown effects in preventing infections of viral origin [21,65,66], and bacterial origin, including those caused by *Staphylococcus aureus* [67]. The main mechanisms behind the antiviral activity of the reported algal SFs have been speculated to be through the binding properties of the sulfated glycans to positive regions of the viral particles during the adsorption period [21,65,66]. This SP-bonded virus particle loses its virulence in attaching to the host cells. Thus the consequent internalization of the virus particles, which would ultimately leave the infection progression, is blocked. As far as we know, no clear structure-function relationships have been achieved regarding this pharmacological activity. Nonetheless, it has been reported that certain structures may show more effectiveness in viral inhibition than others [21]. Although there is some speculation that marine SFs and SGs can act as antiprotozoal or antifungal agents, no solid evidence exists so far supporting these activities. Their antibacterial action is also unclear as well.

## 2. Conceptions in Fucanomics and Galactanomics

Fucanomics and Galactanomics would be defined specifically as international subprojects of glycomics, comprising a systematic, compiled body of research about the structures, functions and metabolic paths involving SFs and SGs, respectively. This should be done using as many different species as possible. The characterization, notation and deposition of the structures would result in a large library of bioactive glycans from the sea. Based on the current definition of glycomics, this project about structural notation related to scientific names of the respective species adequately fits into the field of glycomics [1].

As detailed below, the intense research and publication of biomedical properties and mechanisms of SFs and SGs would represent a large, if not the most valuable, task of Fucanomics and Galactanomics. Additionally, an understanding about the regulative mechanisms involved in the biosynthesis of SFs and SGs, and the consequent impact of such mechanisms on the structural features, and in turn on the resultant biomedicinal properties of these glycans, would comprise another important objective of research. In fact, some reports concerning structural and functional changes influenced by either environmental or biological conditions have already started to emerge [68,69,70,71].

Annual seasons (winter *vs.* summer) have been demonstrated to be influential in sulfation patterns of sea urchin SFs, and thus a hypothetical controlling season-related mechanism in reproducing this invertebrate was raised [69]. Seasonal changes were also documented to be influential in structures of algal SFs and SGs, but still with no clear reasons [70]. The changes in sulfation density and consequently in both structural and functional properties of SGs in marine angiosperms were recently reported to be directly coupled to variations in salinity levels of the estuary regions which these organisms naturally inhabit [68]. Metal contamination in polluted areas has been shown to be influential in the expression of algal SPs, and hence in their respective abundance in cell walls [71]. These two last observations raise speculation about sulfation content of these glycans, somewhat related to the balance of counter-ions in the environment in which these polymers have been synthesized. In any event, unveiling these influential mechanisms associated with the task of deciphering the unknown biosynthetic routes of SFs and SGs would consequently give to Fucanomics and Galactanomics the proper knowledge to better handle and explore the beneficial properties of SFs and SGs. This is the rational research conduct in drug development based on naturally occurring carbohydrate-based molecules that might show possible structural variations dependent on external factors. The achievements from delineated researches might provide a boost to the current medicinal *status-quo *of SFs and SGs.

## 3. What Make Fucanomics and Galactanomics Special Glycomics Subprojects?

At first glance, the full structural assignment of an over 400 residue-complex glycan would appear to be an impossible mission for any glycoscientist. However, structural patterns based on oligomeric repetitions (Table 2) can greatly reduce the obstacles in structural studies involving complex carbohydrates. This regular pattern makes the structures of long glycans more manageable to common analytical practices, thus facilitating data interpretation [15,17]. Even though the glycans listed in Table 2 apparently show some relative structural simplicity, the diversity in structural forms and sulfation patterns is still high. Such variety still keeps both the species-specific identity and the capacity of these glycans to achieve biological actions through their structural “codes” [15]. This was initially and successfully accomplished in data interpretation of sea urchin fertilization through species-specific acrosome reactions [15,16,36,37] It seems to be similarly useful to understand the differential effective levels in medicinal properties of SFs and SGs [15,16].

Among naturally occurring bioactive high-MW SPs described so far in glycobiology, GAGs for example, SFs and SGs are even more unique in terms of structural patterns, together with their potent medicinal properties, from the viewpoint of achieving accurate structure-function correlations [15,16]. The consequent accuracy in structural-biofunction relationships can be exemplified by the amplified anticoagulant effect of SFs carrying lower proportions of Fuc*p* residues, exclusively 2-*O*-sulfonated [15,16].

It is worth highlighting that the majority of the work undertaken so far to study the biomedical properties of marine SFs and SGs (section 1.4), has used molecules extracted from algae with a high degree of heterogeneity [46,47,48,49,50,51,52,53,54,55,56,57,58,59,60,61,62,63,64,65,66,67]. Although algal molecules may reach great levels of effectiveness in certain assays, their structure-function correlations are more difficult to determine. On the other hand, the use of well-defined SFs and SGs from invertebrates, and a few red algal species (Table 2), have enabled more confident propositions [15,16]. This places Fucanomics and Galactanomics as quite distinct subprojects as compared to other “glycan-omics”, especially for those that may show therapeutic action [3,5,6,7,8,9,10,11]. It also comprises a huge step of glycomics towards the next pre-clinical testing of carbohydrate-based drug candidates, once potential bioactive glycans can be presented together with their respective biomedical mechanisms, and accurately studied through reliable structure-biofunction relationships.

## 4. The Impact of Drug Development in Glycomics

Glycomics would lose some of its major impact if the medicinal uses of exogenous glycans were left aside. In the case of GAG molecules, however, the importance of such therapeutic actions, of carbohydrate-based molecules has already been discussed previously [7]. It is worth remembering that GAG heparin is the second most used (to peptide insulin) natural macromolecule in medicine. Heparin naturally occurs in mast cells, and seems to have a primary key role in inflammation and allergic reactions [72]. However, due to its great affinity to the thrombin-inhibitor, antithrombin [73], unfractionated heparin and its low-MW derivatives are clinically explored for stopping the clotting processes in patients undergoing extracorporeal circulation during surgery or renal dialysis [74]. The low molecular variants of heparins also serve as the primary treatment of deep vein thrombosis, since these molecules contain more antithrombin-binding motifs per chain unit. As a consequence of the high incidence of thromboembolic disorders, especially in developed countries, there is a large-scale production of heparins. But due to some side-effects of this drug, new alternative anticoagulant agents are needed (Section 1.4.2) [15,16]. Anticoagulant effects have also been observed for some SFs and SGs, although at lower potencies than heparins, but clearly with lower side-effects also (Section 1.4.2). In addition, as opposed to heparin that can exhibit a high risk of contamination because of its mammalian origin, the SFs and SGs present an additional advantage over this GAG type: they are basically isolated from marine organisms which results in a much lower incidence of contaminants [15,16,50]. Such clinical benefits of SFs and SGs in anticoagulation likely in other biomedical functions (Section 1.4) are relevant contributing elements from Fucanomics and Galactanomics as from glycomics. The development of drugs based on natural occurring carbohydrates should be one of the greatest objectives of these projects, mainly because of their direct implications to human health.

## 5. Future Approaches of Fucanomics and Galactanomics

Because of the relatively recent description of SFs and SGs, many tasks have yet to be accomplished in Fucanomics and Galactanomics. One would be the prompt depositing of structures already characterized (Table 2) in an informative public carbohydrate databank. The deposition of structures related to their scientific names would end up in a valuable library of potential biomedical glycans to be shared with many laboratories in the world, thus speeding up data generation and collaborative networks regarding the science of SFs and SGs.

Another task would be research on the biosynthetic mechanisms of SFs and SGs. As opposed to GAGs, in which all steps are well-known at such a level that enables even proper manipulation of the biosynthetic systems for different purposes [75], the biosynthesis pathways of SFs and SGs are virtually unknown. Although some information about sea urchin (sulfo)transferases [76] and precursor nucleotide-sugars may exist, the biosynthetic routes of these glycans seem still to be unknown. Clarification of the abiotic and biotic determinants in molecular biology of SFs and SGs comprises another fruitful research line, as described in section 2.

Another task would be an attempt to establish some concise phylogenetic relationships between SFs and SGs. For instance, up to now, only linear SFs or SGs have occurred in echinoderms. In echinoidea (sea urchins), the structures are limited to a very short set of characteristics: only 2-*O*- and/or 4-*O*-sulfonation, together with a few types of glycosidic bonds (either α(1→3), or α(1→4), or β(1→3)) [15,16]. This observation raises the hypothesis of a possible short number of (sulfo)transferases in these invertebrates and the concept of very organized or simpler biosynthetic machinery as opposed to those of mammal SPs, like GAGs. In the latter case, a higher number of anabolic enzymes (including isoforms), is the largest contributing source for the consequent structural heterogeneities [77]. In a previous report, we have established a tentative phylogenetic view for SGs [15,16,21]. We noticed that chains of 3-linked β-Gal*p* are heavily conserved throughout marine taxonomic groups (including red and green algae, sea angiosperm, clams, sea urchins, and tunicates), with a strong tendency toward 4-*O*-sulfonation in algae and marine angiosperm, 2-*O*-sulfonation in invertebrates, and 6-*O*-sulfonation, more randomly distributed among various organisms. This indicates that galactosyltransferases seem to be preserved through marine species, but sulfotransferases are likely to be more specifically distributed. Although 4- and 2-sulfotransferases are hypothetically considered to be preserved in plants and in invertebrates, respectively, 6-sulfotransferases are broadly dispersed among marine organisms. This phylogenetic assumption is quite preliminary and no correlation with SF structures has been undertaken so far. It is worth mentioning that these phylogenetic points are just speculative because they were based solely on the structural characteristics of glycans. The structural features of SFs or SGs taken alone cannot become the main tools to propose a definitive phylogenetic relationship if gene expression, transcription levels and amounts of nucleotide-sugar precursors and anabolic enzymes are not considered. Hence, this phylogenetic study is intrinsically dependent on the researches about the biosynthetic routes of SFs and SGs, discussed in the previous paragraph.

The studies concerning the spatial geometry (3D structural view) of SFs and SGs, their thermodynamic behaviors, and their conformational changes upon molecular interactions with the main proteins involved in the diseases documented in section 1.4 (coagulation (co)-factors, selectins, cytokines/chemokines, growth factors, endothelial adhesion-molecules, surface receptors of virus particles and bacteria) would represent outstanding breakthroughs of Fucanomics and Galactanomics. However, the high-MW of these glycans is the main issue for these advanced approaches, and would enhance complexity in both data acquisition and interpretation. Therefore, the production of SF- and SG-derived oligosaccharides is a fundamental primary step. In this direction, we have already established a protocol to efficiently produce low-MW SF-derivatives that still retain both well-defined molecular structures and biological properties [78,79]. Specific experiments of NMR spectroscopy (relaxation rates, NOE-based or residual dipolar coupling techniques) should be able to provide information regarding conformation (with or without the presence of binding proteins) as well as flexibility of these specific marine SPs. Degrees of freedom in studies of molecular motions related to sulfation patterns would help to understand the structural influences in molecular dynamics, as well as the binding capacities in protein interactions during their biomedical roles.

## 6. Inclusion of SFs and SGs as Topics in Forums, Consortiums, and Meetings of Glycomics

In the USA, the working group convened by the Division of Blood Diseases and resources of the National Heart, Lung, and Blood Institute (NHLBI), was created with the primary objective of identifying scientific opportunities and priorities emerging from the recent explosion of technological and biological advances from the glycosciences [80]. Fucanomics and Galactanomics would fit perfectly as topics of this objective. This reunion was a consequence of specific glycoscientific objectives raised by the Consortium for Functional Glycomics, funded by the National Institute of General Medical Science (NIGMS). This consortium was initiated and funded by the US National Institute of Health (NIH). In this report about the roles of glycans in hemostasis, inflammation and vascular biology [80], the following is said: “there is a need for funding mechanisms that will bring together the existing experts in the study of glycans with investigators in blood and vascular diseases, to foster fruitful collaboration that will bridge the existing gulf. Importantly, this frontier area is also ripe for translational research and drug development. It is also noteworthy that this situation is different in many European and Asian countries, e.g., Sweden, Australia, and Japan, where there are major nationwide commitments to, and interest in, the study of glycans. Thus, the USA lags behind the rest of the world in taking advantage of these opportunities”.

Here, we want to reiterate that Brazil might even lag behind the USA in terms of creating these glyco-organizations and in taking the proper advantages of its own national glycoscientific opportunities. This can be seen by the lack of an official consortium in Brazil specifically concerning glycoscience. As documented here though, it is noteworthy that the majority of the results obtained with SFs and SGs of well-defined structures (Table 2), and with promising medicinal activities, were conducted by Brazilian scientists in Brazil! Furthermore, most of the marine species from which these novel SPs were extracted, were collected along the sea-shores of this country (Table 2).

Through this publication, we want to express the high importance in including these new glycans (Table 2) into either National or International Organization(s), in order to prompt the progress of Fucanomics and Galactanomics via possible collaborative and growing research networks. This should be undertaken with high priority, always keeping in mind the main objective of these glycomics subprojects, which is to provide to human society the right and proper medicinal benefits from SFs and SGs, especially those involved in hemostasis, inflammation and vascular biology, as pointed out by the committee of the above-mentioned glyco-forum report [80]. SFs and SGs exhibit great effectiveness in these three systems mentioned (section 1.4), and must be a topic in this or other glyco-forums as well.

## 7. Concluding Remarks

Here, we have made clear the importance of Fucanome and Galactanome to glycomics. This subdivision would make SFs and SGs more well known and participative within glycobiology. By definition, Fucanomics and Galactanomics comprise the full science related to the specific marine glycans composed mostly of sulfated Fuc*p* and Gal*p* units. These glycans show a broad range of medicinal functions and thus would serve as valuable carbohydrate-based therapeutic candidates in the science of drug discovery and development. Their pharmacological actions cover many systems, including inflammation, vascular biology, oncology, oxidative stress, virosis, pathogenesis, among other possible systems not discussed here. The significance of these therapeutic applications of Fucanomics and Galactanomics is highly relevant in order to enhance the glycomics impact, and its international recognition and importance. The rare structural features of some SFs and SGs (those composed of well-defined sulfation patterns and regular repeating oligomeric units, Table 2) make Fucanomics and Galactanomics unique glycomics subprojects because of the facilitated correlation of the biomedicinal levels achieved with the structural properties of SFs and SGs. The establishment of these structure-biofunction relationships has been more difficult to accomplish using other native glycans, including GAG polymers, but conversely have proved to be quite feasible using SFs and SGs.

It has also become clear that even though the division of glycomics into subprojects is really demanding and necessary, the complexity of studying such subprojects still remains high. There is a high chance in the future for assuming Agaranomics and Carrageenomics as hierarchically-related subprojects of Galactanomics here proposed. This would raise the complexity of Galactanomics, but would make easier the organization of large amounts of data. Possible other “glycan-omics” about to show up are Chondroitinomics, Heparinomics, Dermatanomics. All these are hierarchically included in Glycosaminoglycanomics, and in turn, in Proteoglycanomics as well.

Empirically speaking, Glycomics turned out to be undertaken more as a set of isolated research lines than just a single ongoing project. Even taking this subdivision trend, the subprojects themselves are quite challenging. By analogy to what was previously mentioned: “The Sialome—Far more than the sum of its parts” [11], here we reiterate: “The Glycome—Bigger than the division of its total”. This division has been shown necessary for Glycomics’ own evolution!
